# Anti-protein glycation and free-radical scavenging properties of Sri Lankan antidiabetic medicinal plant *Salacia reticulata* l. (Kothala Himbutu)

**DOI:** 10.1186/s12906-023-04169-4

**Published:** 2023-11-03

**Authors:** Galbada Arachchige Sirimal Premakumara, Walimuni Kanchana Subhashini Mendis Abeysekera

**Affiliations:** 1 Department of Basic Sciences & Social Science, Faculty of Nursing, University of Colombo, Colombo, 00300 Sri Lanka; 2 Industrial Technology Institute, 363, Bauddaloka MW, Colombo, 00700 Sri Lanka; 3 Department of Agricultural Technology, Faculty of Technology, University of Colombo, Colombo, 00300 Sri Lanka

**Keywords:** *Salacia reticulata*, Glycation, Diabetic, AGEs, Medicinal plants, Sri Lanka

## Abstract

**Background:**

Decoctions of the root and stem of the medicinal plant *Salacia reticulata* is an indigenous remedy for diabetics and its complications in Sri Lanka. In diabetics, the formation of advanced glycation end products (AGEs) leads to many pathologies. Nevertheless, the anti-protein-glycation property of this plant is poorly documented. This study reports the anti-protein-glycation and radical scavenging potential of various plant parts of *S. reticulata*.

**Methods:**

Hot water extracts (2g dried powder/50 ml) of root, stem, leaf, twigs, and fruits at various concentrations (15.6 to 500.0 µg/ml) were subjected to anti-glycation and glycation reversing assays in vitro. 1,1-diphenyl-2-picrylhydrazyl (DPPH) assay was used for free radical scavenging property.

**Results:**

Various plant parts of *S. reticulata* showed anti-protein-glycation and free-radical scavenging activities. IC_50_ for the anti-glycation activity of root, stem, leaf, twigs, and fruit extracts were 11.92 ± 1.14, 35.18 ± 2.79, 113.3 ± 1.91, 149.59 ± 1.06, and 1120.37 ± 229.48 µg/ml respectively. IC_50_ of Rutin was 21.88 ± 2.82 µg/ml. EC_50_ of the root, stem, twigs, and leaf extracts for glycation reversing was 102.09 ± 6.23, 116.99 ± 5.82, 154.45 ± 5.79, and 278.78 ± 14.19 µg/ml respectively. The EC_50_ values for the radical scavenging activity of leaf, stem, and roots were 26.4±4.7, 9.0±1.2, and 9.1±1.3 respectively. Root had significantly (*p<*0.05) high activity for all the parameters tested.

**Conclusion:**

*Salacia reticulata* possess anti-glycation, glycation-reversing, and free radical scavenging activities. Other than root and stem, the leaves and twigs too may be a useful source for anti-diabetic bioactive molecules.

## Background

Diabetes is in epidemic proportions throughout the world [1–3]. At present, 537 million people are suffering from diabetes mellitus worldwide and the numbers are projected to be increasing to 643 million by 2030, and 783 million by 2045 [4].

Diabetes is characterized by persistent hyperglycaemia and attributed to malfunctioning in insulin secretion, resistance to insulin peripheral actions, or both [5, 6]. Chronic hyperglycaemia leads to the formation of AGEs in body tissues [7–9]. Formation of AGEs subsequently can lead to protein cross linking and render development and progression of several diabetic related complications: arterial wall stiffening, vascular damage, low myocardial compliances, neuropathy, cataracts, impaired wound healing [8–12], neurological complications and aging [7]. The prevention of AGE formation and reversing will prevent these complications and help in the management of this chronic disease. Therefore, glycation inhibitors and glycation reversing agents are of urgent need in the prevention and management of diverse disease conditions.

Genus *Salacia* is distributed throughout the tropics and *S. reticulata* L. is found in the submontane forests in Sri Lanka and India. *S. reticulata* (Syn Kothala Himbutu) is predominant in Sri Lanka while *S. oblonga* and *S. chinensis* are also present in Sri Lankan forests. In ayurvedic and indigenous medicines, this plant is used in the treatment of diabetes and several other conditions [13–16] and in Sri Lanka, decoctions made out of the root and the stem are used in the traditional formulations [13, 16, 17]. The anti-diabetic activity of the roots and stems of this plant has been scientifically validated using in vitro, in vivo*,* and clinical studies [18–24]. Nevertheless, only very few studies have shown that bark and root extracts can reduce glycated hemoglobin HbA1c [18, 19]. Further, there are no studies on the effect of leaves and twigs of *S. reticulata* on any anti-diabetic properties. The present study was conducted to study the effects of *S. reticulata* root, stem, leaf, twigs, and fruit extracts on protein glycation in view of the sustainable exploitation of this medicinal plant in therapeutic and preventive applications. Further, since there are no comparative studies on the anti-oxidant strength of root, stem, and leaf extracts of *S. reticulata*, we studied the free radical scavenging activity using DPPH radical scavenging assay.

## Materials and methods

### Materials

#### Plant material

*S. reticulata* fruit, leaves, twigs, stem, and roots were collected from a privately managed *Salacia* plantation in Naththandiya, Sri Lanka. Authentication of the plant species was carried out by Dr.R.M.Dharmadasa of the Herbal Technology Section of Industrial Technology Institute, Sri Lanka with assistance from National Herbarium, Peradeniya, Sri Lanka. A voucher specimen is deposited at the Herbal Technology Section of Industrial Technology Institute, Sri Lanka (Code number: HTS/23276). The experimental research and field studies on plants, including the collection of plant material, comply with relevant institutional, national, and international guidelines and legislation.

#### Chemicals and reagents

Bovine serum albumin (BSA), D-glucose, trichloroacetic acid (TCA), and DPPH were purchased from Sigma-Aldrich, USA. All the other chemicals used for the preparation of buffers and solvents were of analytical grade.

#### Preparation of hot water extracts

The collected plant materials were washed with distilled water to remove any extraneous matter and air-dried in an air-conditioned room (25 ± 2 °C) for 6 days. Then, the dried plant parts were separately ground into fine powders using a laboratory grinder. Powdered root, stem, leaves, twigs, and fruit samples were subjected to hot water extraction (2g in 50 ml of distilled water for 20 min), filtered, centrifuged (at 6000 rpm for 10 min) and the supernatants were lyophilized (Christ-Alpha 1-4 Freeze dryer, Biotech International, Germany) and used in the anti-glycation and glycation reversing assays (fruits were not tested in glycation reversing assay). Part of the supernatant was used for the free radical scavenging assay.

## Methods

### Anti-glycation assay

The anti-glycation assay was performed according to the method of Matsuura *et al*. (2002) [25] with some modifications. Lyophilized extracts of *S. reticulata* root, stem, leaf, twigs, and fruits (*n=*3 each) at 5 different concentrations (15.6, 31.2, 62.5, 125.0, and 250.0 µg/ml) were used in the assay. Reaction mixtures containing 800 µg BSA, 400 mM glucose, and different concentrations of *S. reticulata* extracts in a reaction volume of 1 ml in 50 mM phosphate buffer (pH 7.4) containing 0.02% sodium aside (w/v) were incubated at 60 °C for 40 h. After cooling, aliquots of 600 µl were transferred to 1.5 ml Eppendorf tubes and 60 µl of 100 % (w/v) TCA was added, stirred, centrifuged at 15,000 rpm at 4 °C for 4 min and supernatants were removed. The resulting AGEs-BSA precipitate was dissolved in 3 ml of phosphate buffer saline (pH 10) and fluorescence intensity was measured at an excitation wavelength of 370 nm and emission wavelength of 440 nm using a spectrofluorometer (Amino-Bowman^®^, Thermo Spectronic, USA). Rutin was used as the standard (positive control). Anti-glycation activity (inhibition %) of each *S. reticulata* extract and rutin was calculated using the following equation.

1 $$\mathrm{Inhibition\;}(\mathrm{\%}) = [({\mathrm{F}}_{\mathrm{c}}-{\mathrm{F}}_{\mathrm{b}})-({\mathrm{F}}_{\mathrm{s}}-{\mathrm{F}}_{\mathrm{sb}}) / ({\mathrm{F}}_{\mathrm{c}}-{\mathrm{F}}_{\mathrm{b}})] * 100$$

Where, F_c_ is the fluorescence of incubated BSA-glucose (control), F_b_ is the fluorescence of incubated BSA alone (blank), Fs is the fluorescence of the incubated BSA-glucose with *S. reticulata* extracts or the positive control (rutin) and F_sb_ is the fluorescence of incubated BSA with the *S. reticulata* extracts or the positive control.

### Glycation reversing assay

The glycation reversing assay was conducted according to the method described previously by Premakumara *et al.* (2013) [26]. A reaction mixture containing 800 µg BSA and 400 mM glucose in 1 ml of 50 mM phosphate buffer (pH 7.4) containing 0.02 % sodium aside (w/v) was incubated at 60 °C for 40 h. After cooling, aliquots of 600 µl were transferred to 1.5 ml Eppendorf tubes, and 60 µl of 100 % (w/v) TCA was added, stirred, centrifuged at 15,000 rpm at 4 °C for 4 min and supernatants were removed. The resulting AGEs-BSA precipitates were dissolved in 50 mM phosphate buffer (pH 7.4) and added with *S. reticulata* extracts ( 15.6, 31.2, 62.5, 125.0 and 250.0 µg/ml; *n=*3 each) in a final reaction volume of 1 ml for incubation at 60 °C for 40 h. After cooling, 60 µl of 100 % (w/v) TCA was added, stirred, and centrifuged at 15,000 rpm at 4 °C for 4 min. The resulting precipitates were dissolved in 3 ml of phosphate buffer saline (pH 10) and fluorescence intensity was measured at an excitation wavelength of 370 nm and emission wavelength of 440 nm using a spectrofluorometer. Percentage glycation reversing was calculated using the following equation.

2 $$\mathrm{Glycation\;reversing\;}(\mathrm{\%}) = [({\mathrm{F}}_{\mathrm{c}}-{\mathrm{F}}_{\mathrm{b}})-({\mathrm{F}}_{\mathrm{s}}-{\mathrm{F}}_{\mathrm{sb}}) / ({\mathrm{F}}_{\mathrm{c}}-{\mathrm{F}}_{\mathrm{b}})] * 100$$

Where, F_c_ is the fluorescence of incubated BSA-glucose (control), F_b_ is the fluorescence of incubated BSA alone (blank), Fs is the fluorescence of the incubated BSA-glucose and *S. reticulata* extracts and F_sb_ is the fluorescence of incubated BSA with the *S. reticulata* extracts.

### Free radical scavenging assay

Five different concentrations, 1.0, 5.5, 15.0, 56.0, and 94.0 μg/mL from leaf extracts and 0.2, 2.0, 8.0, 15.0 and 31.0 μg/mL from stem and root extracts were used in the assay. DPPH (1 mM in methanol) stock was prepared and stored in an amber coloured bottle. DPPH working solution was prepared by mixing 100 μl of stock DPPH solution with 400 μl methanol in 1 ml of the final assay. Positive control was run without samples and negative control was run in triplicate without DPPH for each concentration of samples. Vitamin E was used as the reference standard. Absorbance was recorded at 517 nm after 15 min. incubation at room temperature (25±2°C). The percentage of reduction of DPPH or quenching percentage was calculated based on the following formula (Molyneux, 2004):

$$\mathrm{Q }= 100 ({\mathrm{A}}_{\mathrm{o}}-({\mathrm{A}}_{\mathrm{c}}-{\mathrm{A}}_{\mathrm{b}}))/{\mathrm{A}}_{\mathrm{o}}$$

Where; Q = % reduction of DPPH; A_o_ – Mean absorbance of control; A_c_=Absorbance of sample; A_b_=Mean absorbance of sample blank without DPPH.

### Statistical analysis

Data represented as Mean ± SEM. Data of each experiment were statistically analyzed using SAS version 6.12. One way analysis of variance (ANOVA) and the Duncan’s Multiple Range Test (DMRT) were used to determine the differences among treatment means. *P <* 0.05 was regarded as significant. Five-point IC_50_ & EC_50_ were calculated using probit analysis.

## Results

### Anti-glycation effect of S. reticulata

**Fig. 1:**
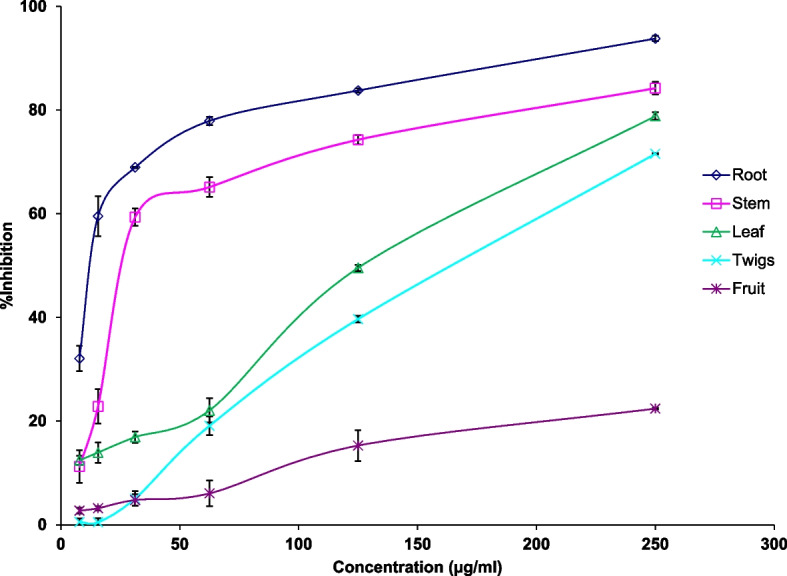
Percentage inhibition of glycation by hot water extracts of root, stem, twigs, leaf, and fruit of *Salacia reticulata*

Anti-glycation activities of *S. reticulata* root, stem, leaf, twigs, and fruit extracts are given in Table 1 and depicted in Fig. 1. All tested extracts of *S. reticulata* showed anti-glycation activity in a dose-dependent manner. IC_50_ values for the anti-glycation activity of root, stem, leaf, twigs, and fruit extracts were 11.92 ± 1.14, 35.18 ± 2.79, 113.3 ± 1.91, 149.59 ± 1.06 and 1120.37 ± 229.48 µg/ml respectively. Root extract had significantly high (*P <* 0.05) anti-glycation activity compared to other extracts and positive control rutin (IC_50_: 21.88 ± 2.82 µg/ml). The potency of different parts of *S. reticulata* for anti-glycation activity was root > stem > leaf >twig>fruit.

**Table 1:** Effect of hot water extracts of various plant parts of *Salacia reticulata* on in vitro protein glycation

**Percentage glycation inhibition**						
***Salacia* extract**	**Concentration (µg/ml)**					
	**15.6**	**31.2**	**62.5**	**125.0**	**250.0**	**R**^**2**^
Root	59.55 ± 3.83	68.96 ± 0.13	77.92 ± 0.83	83.79 ± 0.40	93.81 ± 0.52	0.8628*
Stem	22.85 ± 3.32	59.37 ± 1.68	65.14 ± 1.90	74.28 ± 0.87	84.23 ± 1.22	0.8890*
Twigs	0.49 ± 0.82	5.08 ± 1.41	19.10 ± 1.78	39.69 ± 0.65	71.57 ± 0.15	0.9898*
Leaf	13.95 ± 1.96	16.93 ± 1.09	22.07 ± 2.34	49.57 ± 0.61	78.86 ± 0.72	0.9840*
Fruits	3.20 ± 0.40	4.78 ± 1.11	6.07 ± 2.49	15.29 ±2.98	22.42 ± 0.15	0.9656*
**Percentage glycation reversing**						
Root	8.50 ± 1.50	21.73 ±1.83	36.71 ± 2.09	59.09 ± 8.35	71.32 ± 1.54	0.8699*
Stem	5.71 ± 1.81	18.15 ± 2.84	28.72 ± 4.55	52.62 ± 1.94	72.98 ± 4.38	0.9412*
Twigs	8.50 ± 1.50	15.23 ± 3.41	32.14 ± 4.87	45.40 ± 1.73	60.22 ± 1.99	0.9030*
Leaf	2.5 ± 1.74	8.05 ± 2.74	21.00 ± 2.43	32.10 ± 3.12	45.96 ± 0.25	0.9254*

Data represented as mean ± SEM

^*^= *P <* 0.05

### Glycation reversing effect of S. reticulata

**Fig. 2:**
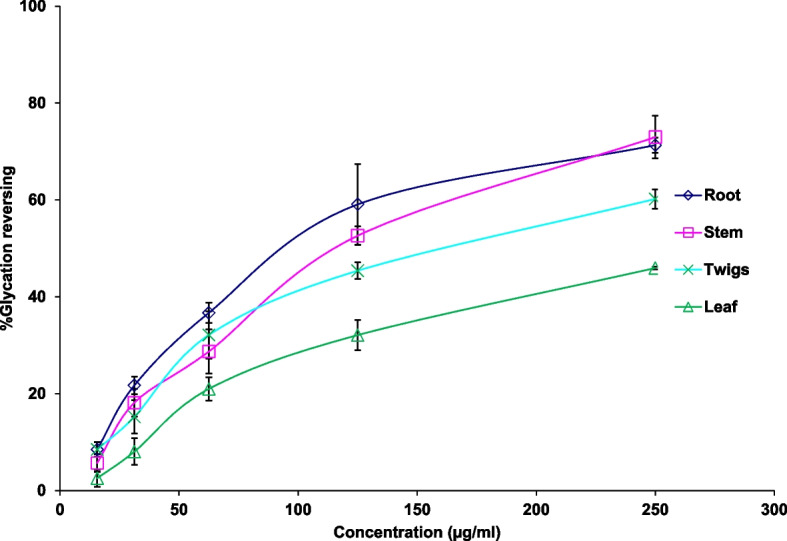
Percentage glycation reversing activity of hot water extracts of root, stem, twigs, and leaf of *Salacia reticulata*

Glycation reversing activities of *S. reticulata* root, stem, leaf, and twigs extracts are given in Table 1 and depicted in Fig. 2. Glycation reversing potential of different parts of *S. reticulata* showed dose dose-dependent relationship. EC_50_ values of root, stem, twigs, and leaf extracts were 102.09 ± 6.23, 116.99 ± 5.82, 154.45 ± 5.79 and 278.78 ± 14.19 µg/ml respectively. The potency of different parts of *S. reticulata* for glycation reversing activity was root = stem >twig> leaf.

### Free radical scavenging effect of S. reticulata

The free radical scavenging property of hot water extracts of *S. reticulta* leaves, stem and root is given in Table 2. The EC_50_ values for leaf, stem, and roots were 26.4±4.7, 9.0±1.2 and 9.1±1.3 μg/mL respectively. EC_50_ of Vitamin E was 4.5±0.5 μg/mL. Percentage free radical scavenging of root and stem remained similar whereas leaf extracts had significantly low (*p<*0.05) free radical scavenging activity compared to stem and root extracts.

**Table 2:** Percentage DPPH reduction by hot water extracts of various plant parts of *Salacia reticulata*

	**Concentration- µg/ml**				
**Salacia extract**	**1.0**	**5.5**	**15.0**	**56.0**	**94.0**
Leaf	0.3±4.7	30.0±3.4	47.1±5.3	92.1±2.5	93.2±1.0
	**Concentration- µg/ml**				
	**0.2**	**2.0**	**8.0**	**15.0**	**31.0**
Root	2.4±3.0	13.5±5.0	46.9±3.3	82.9±5.5	90.5±1.1
Stem	5.7±3.9	17.7±4.8	48.8±4.8	83.8±5.3	91.0±1.0

Data represented as mean ± SEM

## Discussion

Reducing sugar glucose is amply available in the circulation and the body fluids of diabetics. Carbonyl groups of reducing sugars go into a series of complex reactions with amino groups of proteins in the protein glycation reactions which is known as the Maillard reaction. Initially, the amino groups of proteins react with reducing sugars to form Schiff bases followed by their products called Amadori rearrangement products. These Amadori products rearrange further to give a range of end products that are known as Advanced Glycation End products [7]. Some of these end products are intensely coloured molecules with typical fluorescence characteristics [7]. Therefore, in this study, the level of Maillard fluorescence was measured to quantitatively analyze the anti-glycation and glycation-reversing properties of *S. reticulata*.

*S. reticulata* is a well-known anti-diabetic plant in Sri Lankan traditional knowledge and the Indian system of Ayurveda [13]. Anti-diabetic activity of this plant has been explained through a variety of mechanisms. However, anti-diabetic activity with respect to anti-glycation activity has been very poorly documented. In this study, we clearly showed that all parts of *Salacia reticulata* possess anti-glycation activity. Interestingly, root extract had significantly high anti-glycation activity, even compared to the positive control anti-glycation agent, Rutin. Therefore, especially root extracts can be used in the management of AGEs associated chronic diseases. Further, the findings of this study add to the knowledge of the multitude of mechanisms of anti-diabetic activity of this plant.

Glycation reversing is the reversing of already formed AGE cross links. It is an approach to attenuate AGE related complications. Such protein crosslink breakers might be useful as therapeutics for the regulation of complications resulting from diabetes, neurological diseases, and aging [7]. However, only a few AGE crosslink breakers are known to date and there are reports of their limited efficacy in *in vivo* studies [7, 27]. Therefore, it is vital to explore compounds with AGEs reversing ability to manage AGE related complications. The findings of this study clearly showed that all parts of *S. reticulata* possess glycation-reversing activity too. These novel anti-diabetic properties further add value to this traditional medicinal plant as an anti-diabetic plant with multiple mechanisms.

This is the first report of the simultaneous presence of anti-glycation and glycation-reversing activities of this medicinal plant in therapeutic use in the Sri Lankan traditional & Ayurvedic anti-diabetic recipes. Interesting and valuable findings of this study are the presence of anti-glycation and glycation-reversing activities in leaves and twigs as well. Therefore, leaves and twigs which can be repeatedly harvested in short cycles, unlike the root and the stem, may be used as a natural anti-diabetic source with anti-glycation and glycation-reversing properties.

Different AGE inhibitors suppress AGE formation at different stages of glycation. Aspirin inhibits protein glycation at the early stage of the glycation process by acetylating free amino groups of protein rendering blocking the attachment of reducing sugars [28]. The inhibitory activities of vitamin B1 and B6 derivatives such as pyridoxamine and thiamine pyrophosphate [7, 29] have mainly been attributed to their abilities to scavenge reactive carbonyl compounds. We have shown here that *S. reticulata* water extracts possess antioxidant activity. Therefore, the anti-glycation and glycation-reversing activities could be attributed, at least partly, to the anti-oxidant compounds present in *S. reticulata*. However, it is difficult to decide exactly at which stage of the glycation process or in what way the intervention by *S. reticulata* extracts is exerted to reduce the glycation reaction. Further experiments are necessary to identify active compound/s, *in vivo* efficacy, and modes of action.

## Conclusion

*Salacia reticulata* possesses anti-glycation, glycation reversing, and free radical scavenging activities. Roots and stems were the most biologically active parts of the plant. Leaves and twigs, which can be repeatedly harvested in short cycles, unlike roots and stems, can be used as a good natural source with anti-glycation and glycation reversing activities. This is the first comparative study to report the anti-glycation and glycation reversing potential of various plant parts of any Salacia species in the world. Further studies are needed to isolate the respective bioactive molecules.

## Data Availability

The data used and/or analyzed during this study are available from the corresponding author upon request.
